# Psychology Education Reform and Quality Cultivation of College Music Major From the Perspective of Entrepreneurship Education

**DOI:** 10.3389/fpsyg.2022.843692

**Published:** 2022-06-09

**Authors:** Xiaoran Wang

**Affiliations:** College of Art, Tianjin University of Commerce, Tianjin, China

**Keywords:** music education, entrepreneurship education, self-efficacy, gender regulation, dialectical thinking

## Abstract

With the continuous development of the times, music education in primary and secondary schools is constantly innovating in terms of teaching concepts, teaching methods, and implementation methods. The reform of basic music education has aroused the reflection of music teacher education in colleges and universities, and the talent training model of music teachers has become a hot issue that has been widely concerned in the new era. To explore the educational significance and influencing factors of entrepreneurship education in college music education and examine the mediating role of self-efficacy and gender adjustment in entrepreneurial education, based on the analysis of music education and entrepreneurial talent training in colleges and universities, a questionnaire survey, statistics, and data processing were adopted in this work. In this work, a path model was established based on the mediating effect and the moderating effect, and the equation model was tested. The results show that music education has a positive correlation with entrepreneurial self-efficacy and a negative correlation with entrepreneurial intention, which has not been proved to have a certain effect on entrepreneurial intention. Entrepreneurial self-efficacy has a positive correlation with entrepreneurial intention, and the mediating effect between music professional education and entrepreneurial intention has also been proved. Since there are gender differences and certain stereotypes that cannot be ignored in music education learning, gender adjustment also has a certain influence on entrepreneurial self-efficacy based on music professional education, thereby having a moderating effect on entrepreneurial intentions. Entrepreneurship education can improve the comprehensive competitiveness of students. As a result, entrepreneurship education can improve the innovation and entrepreneurship education model and the music education system in colleges and universities by combining with the relationship between entrepreneurial efficacy and intention, and it contributes to the overall cultivation of talent in colleges and universities.

## Introduction

With the development of the concept of quality-oriented education, the demand for comprehensive talent in college education is increasing. While constantly improving their professional knowledge and technical level, students should also pay attention to the ability training in innovation, entrepreneurship, and aesthetic education to improve their comprehensive quality and competitiveness. Entrepreneurship education is one of the important manifestations of educational reform and innovation in colleges and universities. At present, innovation and entrepreneurship education have a clear policy promulgation and task objectives. There are many shortcomings in traditional college music education, and the lack of innovation in the education system makes it difficult for students to improve their innovation ability (Wu and Wu, 2017). The existing educational methods and physical conditions tend to lead to the lack of practical ability and experience of professional students. Music majors and music innovation and entrepreneurship have high requirements on students’ practical ability. Regardless of performance or creation, if there is no strong practical ability, it will lead to the limitation of innovation consciousness, and it is difficult to introduce excellent creation results. Moreover, it is always difficult for students majoring in music to start their own businesses after graduation. The development and progress of entrepreneurship education can play a targeted role in guiding these students (Zheng et al., 2018). Some investigations in related fields have been performed. Under the background of quality education, Jiang (2020) took the basic general education course of music theory in colleges and universities as an example, introduced the necessity of the construction of the basic general education course of music theory in colleges and universities, and analyzed the current situation and existing problems (Jiang, 2020). Wang (2021) discussed the development status, advantages, and specific adoption of computer music technology in music education (Wang, 2021). Furthermore, Markovi et al. (2020) explored several aspects of music education reform in middle schools. The major aspects of the reform of Serbian secondary music schools and their integration with similar schools in the European education system were mainly investigated (Markovi et al., 2020). The purpose of the authors was to recognize some crucial aspects related to curriculum change and content innovation, namely, the textbook and the assessment process. Through the analysis, the core issues of the literature are mainly focused on the specific content of the reform of music teacher education. Nevertheless, there are few discussions on the educational reform and quality training of music majors in colleges and universities from the perspective of entrepreneurship education. Therefore, the related issues from this perspective are studied. The innovative talent training and teaching system in colleges and universities is being gradually improved and implemented, and targeted reforms have been carried out in combination with teaching in various fields of China. The teaching system should not only be scientific and effective but also meet the actual growth needs of students. The music major itself is a professional system related to the field of art. In the process of teaching reform, it is necessary not only to cultivate students’ basic knowledge control ability and application ability but also to build a complete quality education system so that students can further value mining on the basis of musical elements and experience the connotation and ideas to improve self-consciousness and create a good core quality of music. This has become a key research object in the development of the Ministry of Education and the field of music.

Entrepreneurship is one of the important driving forces for economic growth and industrial upgrading. With the arrival of the golden age of entrepreneurship, college students have gradually become the main force of innovation and entrepreneurship because of their professional knowledge reserve, human capital, and young innovative thinking. However, according to the *2020 College Student Employment Report,* only 2.5% of 2019 graduates started their own businesses after graduation compared with 2.9 percent in 2017 and 2.7 percent in 2018. Research has shown that whether graduates are willing to carry out innovation and entrepreneurship mainly depends on their views on entrepreneurship. When personal traits are enhanced through comprehensive learning, the psychology of autonomy will affect the entrepreneurial intention of college students. Such psychology will subtly change the way college students think and behave to effectively compensate for the lack of entrepreneurial talent among college students. Music education is regarded as an important means and way to cultivate and implement all-round talent reserves in international research. In this kind of education, students will not only emphasize the learning of skill-based content but also pay attention to music itself. To cultivate students’ overall humanistic quality, Harvard University takes up a large proportion of the humanities, among which there are 45 “literature and art” courses and 7 music courses (Ravn and Srensen, 2021).

The significance of this work was divided into practical significance and theoretical significance.

Its practical significance lies in the following aspects. Based on the current research and analysis, many colleges and universities regard music education as the main path to improve the aesthetic standards of students, so paying attention to the development of music education in ordinary colleges and universities can help improve the aesthetic education standards of universities and colleges. In recent years, music education in ordinary colleges and universities in China has grown rapidly, and the situation is gratifying, but some colleges and universities still ignore music education when considering the overall development of the school and do not include it in the discipline development plan. Many people’s cognition of it is superficial, and music education does not occupy an important position in college education. Faced with this situation, the author makes a practical reference for the teaching of music education in ordinary colleges and the development of students’ overall quality by investigating and researching the current situation of music education in ordinary colleges and universities.

Its theoretical significance is embedded in the following aspects. Music pedagogy is one of the hottest art disciplines in the world today, and related information can be queried on many platforms. Music pedagogy is a new type of interdisciplinary subject, and its particularity lies in the fact that pedagogy and musicology are two independent subject systems, but the integration of subjects is an inevitable trend on the road of world education development. It is the theoretical pillar of the development of music education in colleges and universities, the necessary basis for the development of music education disciplines, the theoretical guidance in music education in colleges and universities, and the theoretical support for making music teaching in ordinary colleges and universities more complete. Based on the theory of music pedagogy, this work investigates and researches the current situation of ordinary colleges and universities that have opened music courses. In the process of in-depth investigation, the author finds the shortcomings of music teaching in ordinary colleges and universities and gives improvement strategies. This survey is also expected to analyze the data carefully, conduct careful comparative research, and bring the theory into practice to provide valuable information for music education in ordinary colleges and universities.

The background of music education reform and quality training was introduced in the first chapter. In the second chapter, entrepreneurial self-efficacy and gender regulation under music education were discussed, and the corresponding hypotheses were proposed. Then, the proposed hypotheses were verified in the third chapter. In the fourth chapter, the results are summarized, and future research directions are expected.

The contributions and innovations in this work can be divided into two points. The first point is the innovation of the research perspective: this work analyzes the education reform and quality training of music majors in colleges and universities in an innovative manner from the perspective of entrepreneurial entrepreneurship education. The second point is the innovation of research methods: this work explores the relationship between talent training and music education in an innovative manner and analyzes the current shortcomings of music education and entrepreneurship education in colleges and universities. Based on the situation and current situation of music education in ordinary colleges and universities, it analyzes, summarizes, and reflects on it and proposes to establish a scientific concept of music education, formulate standardized teaching content, strengthen the construction of teaching staff, and improve improvement strategies and countermeasures for the education management system.

### Entrepreneurial Self-Efficacy and Gender Regulation Under Music Education

#### Relationship Between Talent Training and Music Education

Music education, an important part of art education, is polarized according to the actual situation of learners and is usually classified as “professional music” education and “general music” education. “Professional music” education is also classified into two types. The first is the cultivation of technical and performance music talents, such as musical instruments, vocal music, music engineering, and songwriting. The other is the cultivation of comprehensive music professional educators. In ordinary colleges and universities, the purpose of music education is generally to promote the aesthetic ability of college students and develop their thinking ability to achieve a comprehensive aesthetic education. Its ultimate goal is training comprehensive talent and improving the competitiveness and social value of university talent.

Music education has its particularity. Its education process does not depend on compulsion but on internal edification. People become soft from the deep heart, willing to accept new things and environment, and the aesthetic diversity will make the heart more open. Music education is not a kind of compulsive mental development education; it will not be mandatory for learners to establish a specific thinking mode and moral standards. Instead, they influence people’s thinking and behavior in a subtle way. People are influenced and changed from deep inside and carry out self-reflection and correction in the process of learning and appreciation.

The ultimate goal of education is educating people, and music education is no exception. With the development of music education, university talent will develop into innovative and thinking talent. Diversified aesthetics will also make the thinking development of college students no longer monotonous and boring. Music, unlike other arts, often fails to convey information intuitively. However, it has a unique form of expression, which requires educators to find ways to let students actively play the imagination in the process. This kind of imagination and creativity is an important part of talent training in colleges and universities. Learning the basis of music theory and music practice can improve the ability of expression, cooperation, and inner hearing. Many music training rehearsals can improve students’ new understanding and ideas about the team. At present, college education usually has no requirements for aesthetics, and this phenomenon often means that it is difficult to improve the aesthetic ability of the educated. With the continuous improvement of the teaching value of musicology, the music teaching system in colleges and universities is also continuously optimized. The original ordinary local colleges and universities have covered musicology majors and music performance, which can meet the actual learning needs of students from the most basic level. At the same time, based on the concept of student-centered and continuous improvement, further strengthening the long-term development of the talent training mechanism for music majors has become a major issue in the development of local colleges and universities.

Aesthetic education plays an irreplaceable role in the process of all-around development of talent in colleges and universities. In recent years, China has been advocating quality education for the people. College music education refers to taking college students as the object of education, the classroom as the teaching form, and theory and appreciation ability as the most basic education. Without music education in aesthetic education, quality education can hardly develop in an all-around way. Music education helps college students improve themselves through its unique education methods. Therefore, music education has gradually become a university that cannot replace other disciplines. The responsibility of music education and the value of the realization of effectiveness are very large so that university talent can achieve a comprehensive transformation and promotion from inside to outside.

To explore the current status of music education in colleges and universities, an electronic questionnaire survey was adopted. Questionnaires and interviews were carried out with students and teachers from four colleges and universities in S City as research subjects, and related literature retrieval and reading were implemented.

#### Entrepreneurial Learning and Entrepreneurial Intention

Entrepreneurial intention, as one of the best indicators to judge entrepreneurial behavior, refers to the psychological state and subjective will of potential entrepreneurs before they pursue their entrepreneurial goals (Zdravi, 2020). According to social learning theory (Zhaleko, 2020), human learning behavior is realized through two aspects: direct experience and observation of the behavior of the demonstrator. Due to the particularity of their group, college students usually lack direct and intuitive entrepreneurial experience. Therefore, the observation of demonstrator behavior will have a more important impact on the entrepreneurial intention of college students. Music education in colleges and universities, as a field of entrepreneurship and basic education of entrepreneurship learning that has not been fully explored at present, means that students have many opportunities to start their own businesses. However, the current status of music education is not sound enough to provide sufficient demonstrator behavior for college students with entrepreneurial intentions (Zhao et al., 2020). As a result, there is insufficient access to information and resource costs. Good and comprehensive music education can provide comprehensive talent reserves for universities. Moreover, it also creates higher competitiveness for college students and an effective and comprehensive springboard for entrepreneurship (Hoffmann et al., 2020) so that students’ feasibility judgment on entrepreneurship can be improved and their entrepreneurial intentions can be stimulated.

Whether college students are willing to engage in entrepreneurship mainly stems from their ideas on entrepreneurship. The understanding of the external environment can be changed to influence entrepreneurial intention through personal characteristics, such as autonomy and innovation enhanced by learning (Watson and Mcgowan, 2019). Comprehensive entrepreneurial knowledge and innovative reserves are conducive to improving the entrepreneurial awareness of college students (Maksimov and Luo, 2021) to stimulate and form entrepreneurial intention. Teaching entrepreneurial knowledge to college students in the form of education is an important means to influence entrepreneurial attitudes and enhance entrepreneurial intentions. In addition, the degree of comprehensive knowledge acquisition is also the key factor influencing intention and idea. Entrepreneurship courses and practice can acquire entrepreneurship knowledge to realize cognitive learning. Gradually comprehensive and practical accumulation through learning can affect the emotion and attitude of entrepreneurship.

The five-level Likert scale (Dalecki, 2019) was used in the work, where the five level means “strongly consistent” and one means “strongly inconsistent.” In music education, six items, including “I can learn practical skills and skills from my current college music course education and use them,” are set up, and the Cronbach coefficient is 0.827, which suggests good reliability.

The questionnaire on entrepreneurial self-efficacy includes the self-efficacy of entrepreneurial management, the self-efficacy of entrepreneurial persistence, and the self-efficacy of entrepreneurial leadership, which is studied in three dimensions (Aimar et al., 2020). The self-efficacy of entrepreneurial management set up four projects, including “I can initiate the design of a business plan and show it to investors.” The self-efficacy set by the entrepreneur insists on four projects, including “I can withstand and cope with the sudden change of the entrepreneurial environment.” The self-efficacy of entrepreneurial leaders was set in four items, including “I support others’ innovative experiments.” Data entry and management analysis were performed *via* SPSS (Statistical Product and Service Solutions) 24.0, and AMOS (Advanced Mortar System) 22.0 was used for statistical analysis of the entered data (Sharma, 2021). Regarding the question of whether there is a common method deviation, two common methods are adopted, one of which is the Harman single-factor test (May et al., 2021).

#### Self-Efficacy and Gender Regulation

Self-efficacy, also known as perceived self-efficacy or sense of self-efficacy, proposed by Bandura in 1977, is used to judge whether an individual has confidence in his or her ability to achieve a certain task. The interaction between the environment, cognition, and other internal elements commonly constitutes an individual’s behavior, and this interaction relationship is also known as “ternary interactive determinism” (Rogoza et al., 2018). The belief of the individual plays a key role in it, and self-efficacy plays a central role in the belief (Lara-Bocanegra et al., 2020). People will have different behaviors when facing the same situation because of their different sense of efficacy. Many foreign studies have shown that self-efficacy in entrepreneurship has a strong ability to judge and predict entrepreneurial intentions (Zhong et al., 2020). The more confident an individual is about his entrepreneurial ability, the higher the entrepreneurial feasibility will be (Zhou et al., 2021). Entrepreneurs with high entrepreneurial self-efficacy firmly believe that they have all the necessary conditions for success (Jarman et al., 2020). Therefore, they prioritized strategies that would make their efforts effective in the face of their history of suffering. Instead, they focus more on their own shortcomings and shift their attention to the possible negative consequences rather than the process.

Some studies have proved that both internal and external factors of the individual and the environment can affect entrepreneurial intention through self-efficacy. According to the research of social cognitive theory (Qian et al., 2018), the interaction among individuals, the environment, and behaviors in the entrepreneurial learning process is integrated. This research discussed the mediating effect of entrepreneurial self-efficacy between education and intention. It was concluded that the comprehensive development of music education is one of the important external environmental factors affecting college students, which can affect students’ entrepreneurial intentions by influencing their entrepreneurial self-efficacy. Self-efficacy plays a mediating role between music professional education and entrepreneurial consciousness (Chen, 2019).

Based on the above research, the following hypotheses are drawn:
H T1: self-efficacy has a positive effect on entrepreneurial consciousness.
H T2: music education can effectively improve college students’ sense of self-efficacy.
H T3: self-efficacy mediates the relationship between music education and entrepreneurial awareness.
H T3a: music education has a positive impact on entrepreneurial intention.

##### Gender Regulation

With the popularization of music professional education, there are some differences between genders in their acceptance ability, emotional expression, behavior, and response to music learning (Wiresna et al., 2020). As the times go by, people’s learning and cognition of music are gradually deepening, and the impression of music gradually turns to the idea that music can stimulate imagination and creativity. In music practice, attention can be focused, coordination of limbs and thinking can be improved (Wu and Song, 2019), and good learning habits can be formed at the same time. However, gender stereotypes still exist in music education and learning. Taking musical instrument education in music education as an example (Wu et al., 2020a), due to the inherent traditional concept, people will think that certain musical instruments are only favored by men. The other is that it is only for women, or that certain musical instruments are just for certain types of people. In this way, learners will not only be greatly reduced, but their thoughts will also be solidified (Yuan and Wu, 2020). Musical instruments do not play the meaning envisioned by their creators, which is not conducive to the realization of the goal of comprehensive, thinking, and creative talent.

However, studies on entrepreneurship education have found that men tend to be more willing to take risks and more likely to be motivated to achieve risks than women. Women, on the other hand, are more likely to choose low-risk options (Lange et al., 2020). What is clear is that women have significantly less favorable entrepreneurial environments than men, with much less social support and opportunities. Therefore, in contrast, men tend to have stronger entrepreneurial intentions (Wu et al., 2020b).

**Figure 1:**
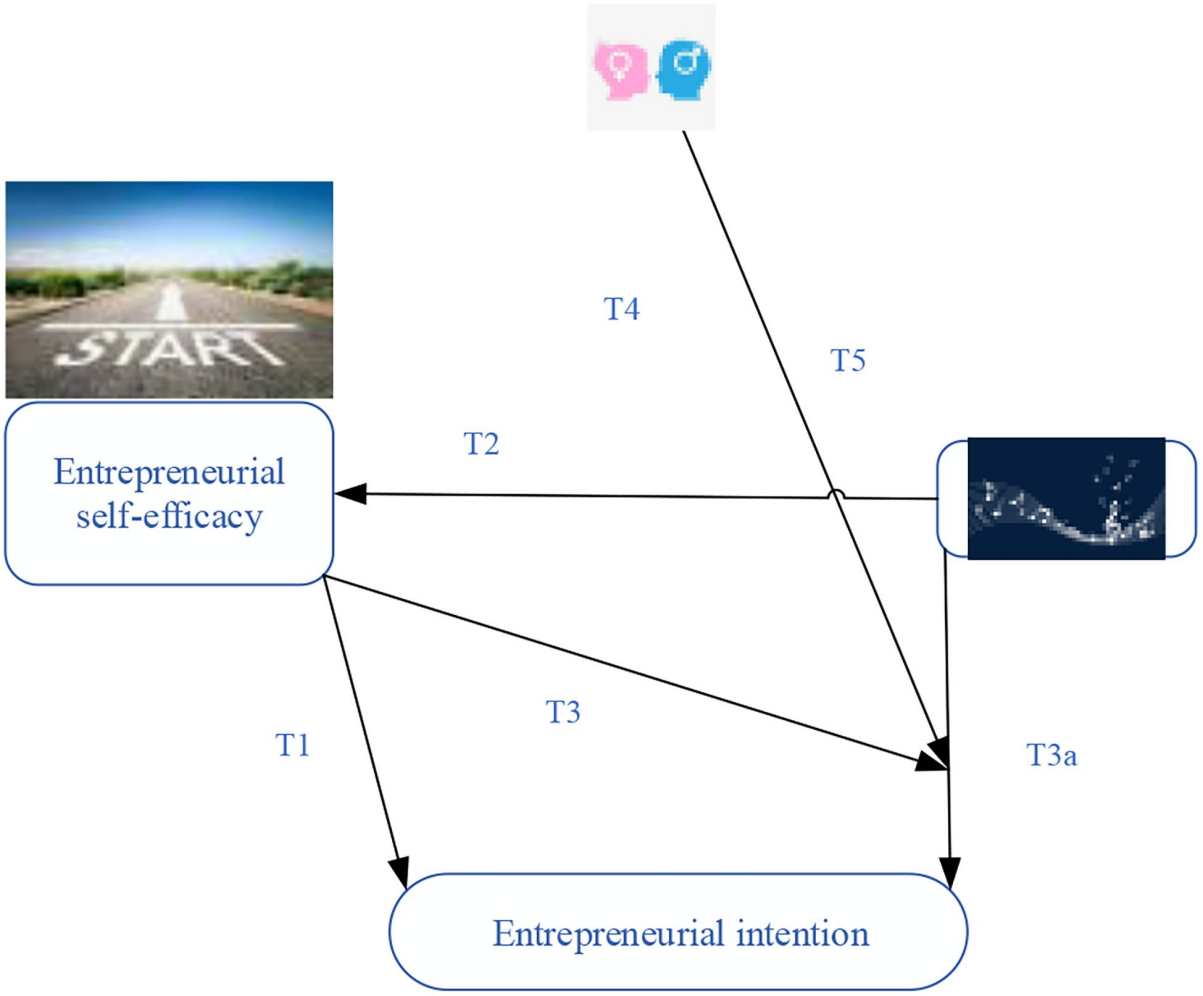
Study model.

Based on the above research, the following hypotheses are drawn:
H T4: gender moderates the relationship between music education and entrepreneurial self-efficacy.
H T5: gender moderates the relationship between music education and entrepreneurial intention.
The proposed model is shown in Figure 1.

It is estimated that music education, as an important part of college learning and life, reasonable reform and quality cultivation of music education can affect college students’ entrepreneurial self-efficacy and thus affect their entrepreneurial intention. However, self-efficacy plays a mediating effect between music education and entrepreneurial intention. The existence of gender also plays a moderating role in self-efficacy, music professional education, and entrepreneurial intention.

### Statistical Estimation Results and Analysis

#### Data Sources

To investigate the current situation of music education in colleges and universities, an electronic questionnaire was used. Students and teachers of four universities in S City were selected as the research objects, and they were asked to receive the questionnaire survey and interview survey. Relevant literature retrieval and reading were implemented. To ensure the diversity and comprehensiveness of the samples and to ensure that the institutions where the research objects were located can basically cover the characteristics of most universities’ music education in S City, 600 undergraduates who were not music majors were selected from 211 universities, ranking A universities, ordinary universities, and private universities. Meanwhile, the thinking mode and aesthetic concept of the objects matured, and they had a certain knowledge reserve and their own understanding of music. The music education teachers from the four universities were selected as the teaching teachers in the survey. There were two sets of questionnaires, including 30 questions. The objects of questionnaire A were the students, and those of questionnaire B were the teachers. The mode of online electronic questionnaires was adopted, which effectively increased the number of questionnaires completed and the efficiency of information collection. In the questionnaire collection, there were 175 questionnaires from 211 universities, 83 from A universities, 192 from ordinary universities, and 120 from private universities, with a recovery rate of 95%. Accordingly, the proportion of female students was 49.7% and that of male students was 50.3%.

### Questionnaire Results and Analysis

The Cronbach coefficients of entrepreneurial self-efficacy were 0.841, 0.837, and 0.740, respectively, showing good reliability. Table 1 shows various adaptation indicators.

**Table 1:** Adaptive indicators of entrepreneurial self-efficacy questionnaire.

Adaptive indicators	Coefficient values
χ^2^/df (Degrees of freedom)	2.979
GFI (Goodness-of-fit index)	0.931
AGFI (Adjusted goodness-of-fit index)	0.882
NFI (Non-normed fit index)	0.915
IFI (Incremental fit index)	0.939
CFI (Comparative fit index)	0.937
RMSEA (Root mean square error of approximation)	0.080

**Figure 2:**
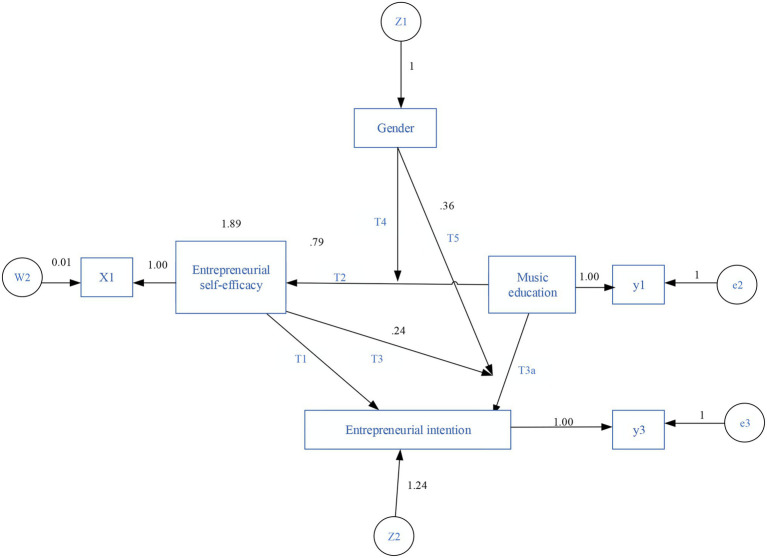
Amos structural equation model constructed here.

The entrepreneurial intention questionnaire was conducted from one dimension, including three items, such as “I have the intention to start my own business after graduation.” Cronbach’s coefficient is 0.909, and the reliability is good. According to the data obtained from the questionnaire, the overall description of entrepreneurial self-efficacy was carried out. According to the questions in the questionnaire, the satisfaction of entrepreneurial self-efficacy was designed to be 1–5. The results are shown in Table 2, and the Amos structural equation model constructed here is shown in Figure 2.

**Table 2:** Overall description of entrepreneurial self-efficacy.

	Self-efficacy of ability	Self-efficacy of behavior	Self-efficacy
Min	1.01	2.01	1.67
Max	4.99	4.99	4.99
*M*	3.6	3.41	3.57
SD	0.71	0.58	0.61

In Table 2, M represents the mean value. The highest score of self-efficacy ability is 4.99, and the lowest score is 0.71. The highest score of self-efficacy of behavior is 4.99, and the lowest score is 0.58. The highest score of self-efficacy is 4.99, and the lowest is 0.61.

After exploratory factor analysis of SPSS 24.0, it was found that the variance seed setting rate of the first factor was within the acceptable range, which was 32.631%. All the scale items are included in the first-order confirmatory factor analysis (Wu et al., 2020c), and the results indicate that the indicators of the first-order confirmatory factor model are not good. The above content shows that the influence of the deviation problem exists, but the research is feasible. The indicators of the first-order confirmatory factor model are shown in Table 3.

**Table 3:** Adaptive indicators of the first-order confirmatory factor model.

Adaptive indicators	Coefficient values
χ^2^/df	8.508
GFI	0.451
AGFI	0.406
NFI	0.459
IFI	0.495
CFI	0.499
RMSEA	0.157

Table 4 shows a survey on the purpose of elective music courses, the degree of knowledge mastery, the degree of preference for teaching methods, and the reasons why college students like music.

**Table 4:** Survey of non-music major undergraduates taking music courses in universities.

Project	Percentage	Project	Percentage	Project	Percentage	Project	Percentage
Personal	31.95%	Master all	1.90%	Very like	2.10%	Influenced by media	22.70%
Credits	35%	Basic	15.80%	Like	30.70%	Influenced by family	2.90%
Optional	21.30%	Partial	57.50%	Sort	58%	Influenced by teachers	2.50%
Improve	11.80%	None	25.30%	Dislike	9.20%	Personal hobby	71.90%

In Table 4, most undergraduates who are not music majors choose music as their optional course because they need to complete credits. Consequently, the non-music major undergraduates do not have much initiative in music courses. Most students can only master a part of what they acquire in optional music courses. Simultaneously, the number of students who do not grasp the content of the courses cannot be underestimated. Currently, the teaching method of music education in colleges and universities is not widely accepted by students. In addition, according to the content of the questionnaires, students’ expectations of teachers’ ability and professional technology accounted for 56.7 and 33.5%, respectively, both of which accounted for a large proportion. However, students have less demand for both the education background and professional title of the teachers.

**Figure 3:**
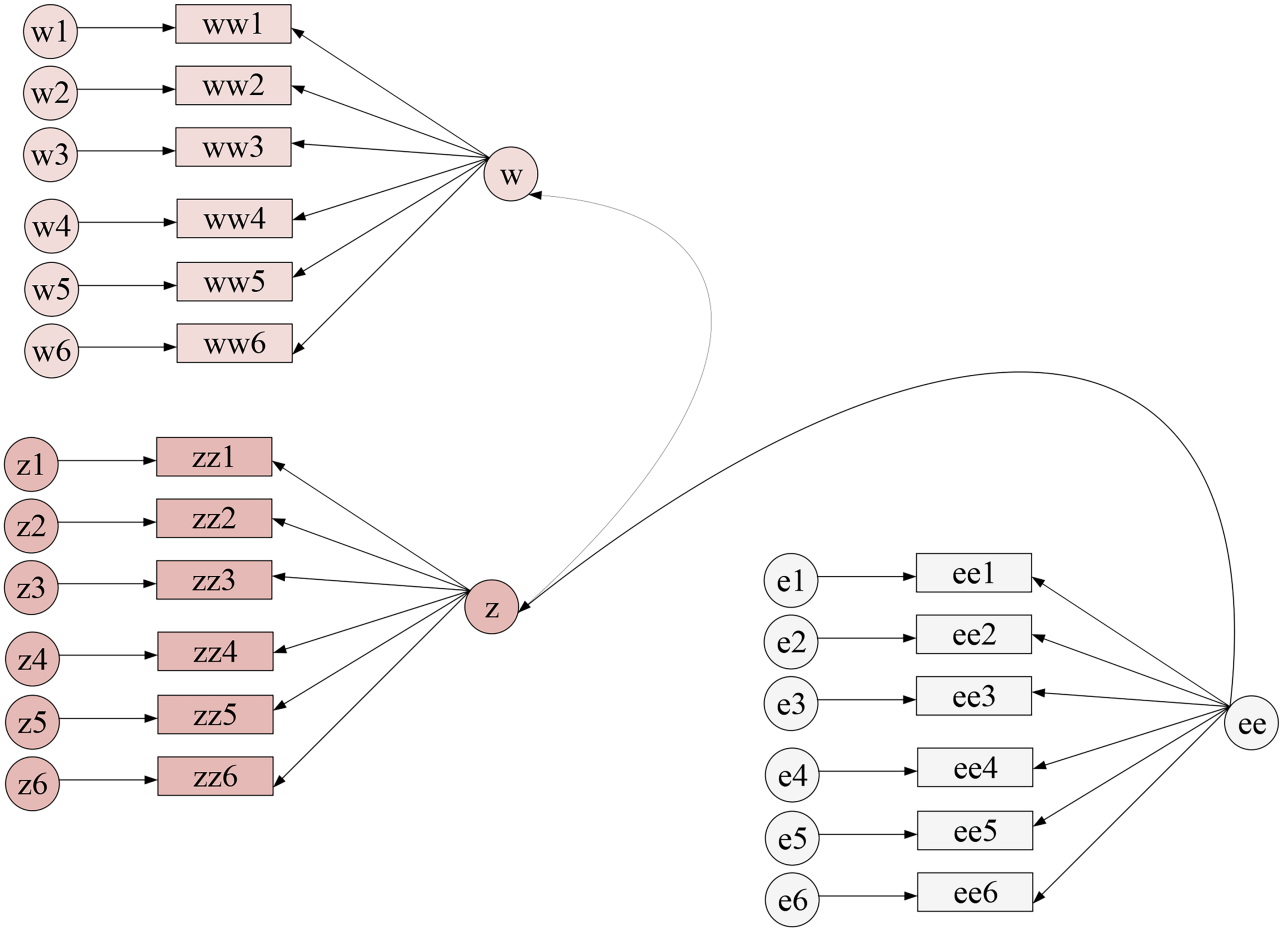
Output result of Amos(1).

**Figure 4:**
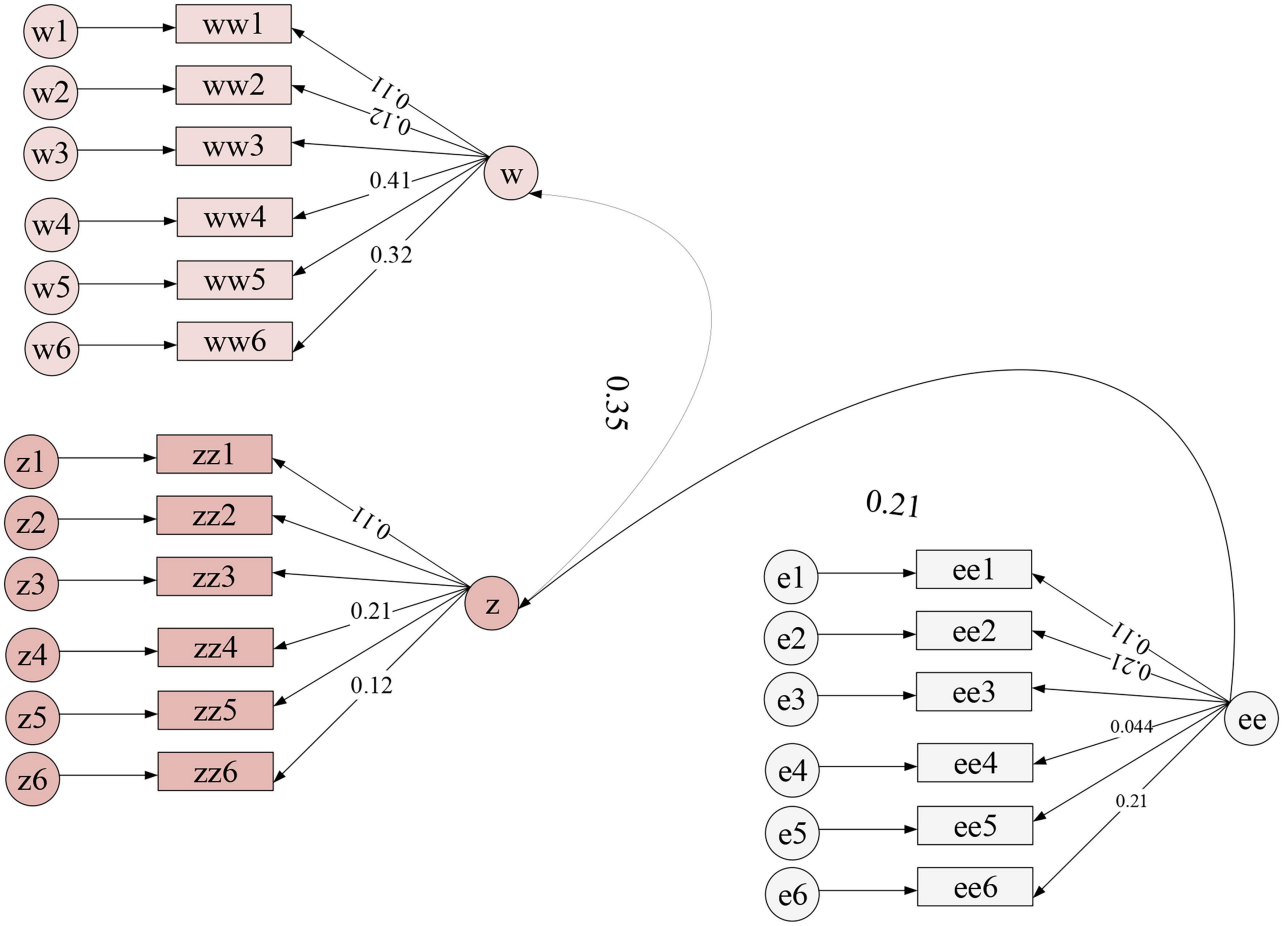
Output result of Amos(2).

SPSS24.0 was used to statistically analyze the mean value, standard deviation, and correlation coefficient of variables, as shown in Table 5. Figures 3, 4 show the Amos output results.

**Table 5:** Fitting index of significance relationship.

Adaptive indicators	Coefficient values
χ^2^/df	2.019
GFI	0.928
AGFI	0.960
NFI	0.932
IFI	0.967
CFI	0.958
RMSEA	0.052

From the correlation analysis shown in Table 5, music professional education and entrepreneurial self-efficacy show a significant positive correlation. There is a significant positive correlation between entrepreneurial self-efficacy and entrepreneurial intention, and music education has a negative correlation with entrepreneurial intention. Using confirmatory factor analysis to compare the mean variances of the test variables, it can be found that the difference between the mean variances of the variables is small, and the variables have good convergent validity.

**Figure 5:**
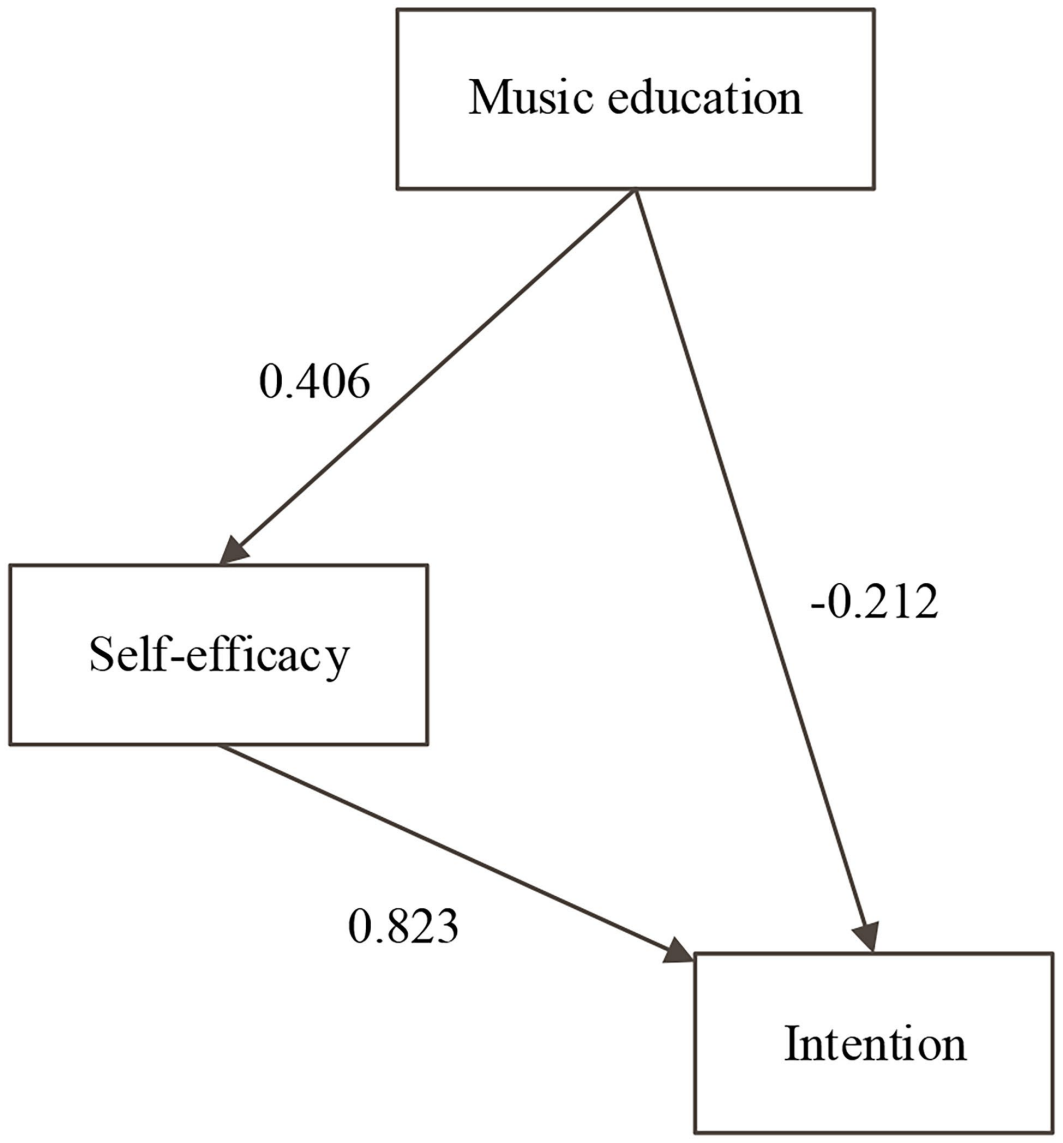
Equation model test.

The structural equation model established in this research takes the acceptance of music professional education as the independent variable, the intermediary variable is entrepreneurial self-efficacy, and the dependent variable is entrepreneurial intention. AMOS 22.0 was employed to test the significance relationship. The equation model test is shown in Figure 5, and the fitting index is shown in Table 5.

According to the results in Figure 5 and Table 5, the values of χ2/df, GFI, AGFI, NFI, IFI, and CFI are all greater than 0.9, and the value of RMSEA is less than 0.8, indicating that the model in this work is feasible. According to the research model in Figure 5, music professional education has a significant positive correlation with entrepreneurial self-efficacy and entrepreneurial self-efficacy on entrepreneurial intentions. Therefore, the hypotheses of T1 and T2 are verified. The influence of music professional education on entrepreneurial intention is negatively correlated, so T3a is not verified. The degree of acceptance of music professional education has a significant and great positive correlation with entrepreneurial self-efficacy, while the negative correlation with entrepreneurial intentions is recessive and has a small impact.

### Analysis of Mediation Effect

**Figure 6:**
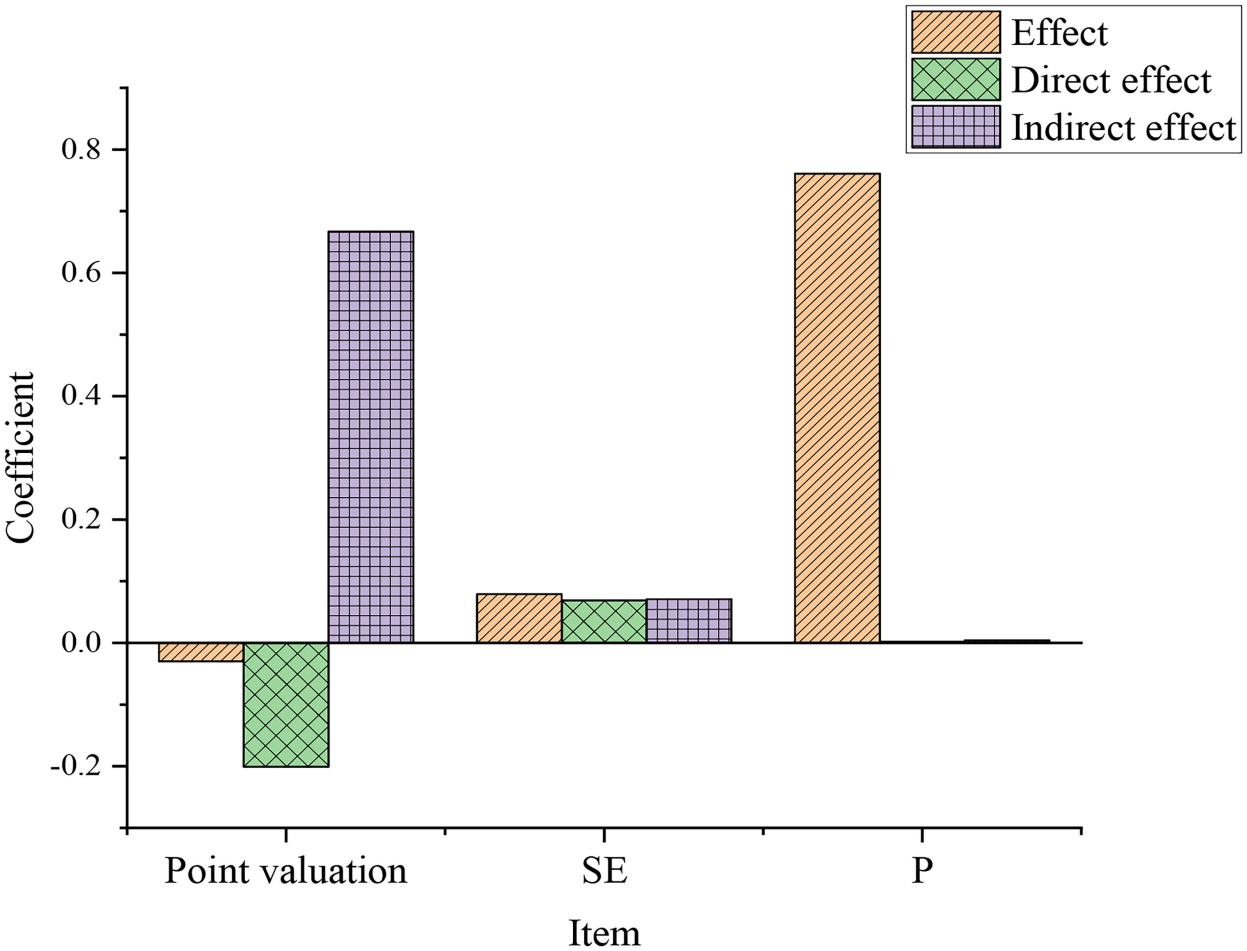
Estimation of the mediating role of music education.

In AMOS 22.0, a total of 300 samples were obtained by random repeated sampling and estimated within a 90% confidence interval. Point estimates, SE (standard error), and P (*p*-value) were obtained. The results of the deviation calibration are shown in Figure 6.

**Figure 7:**
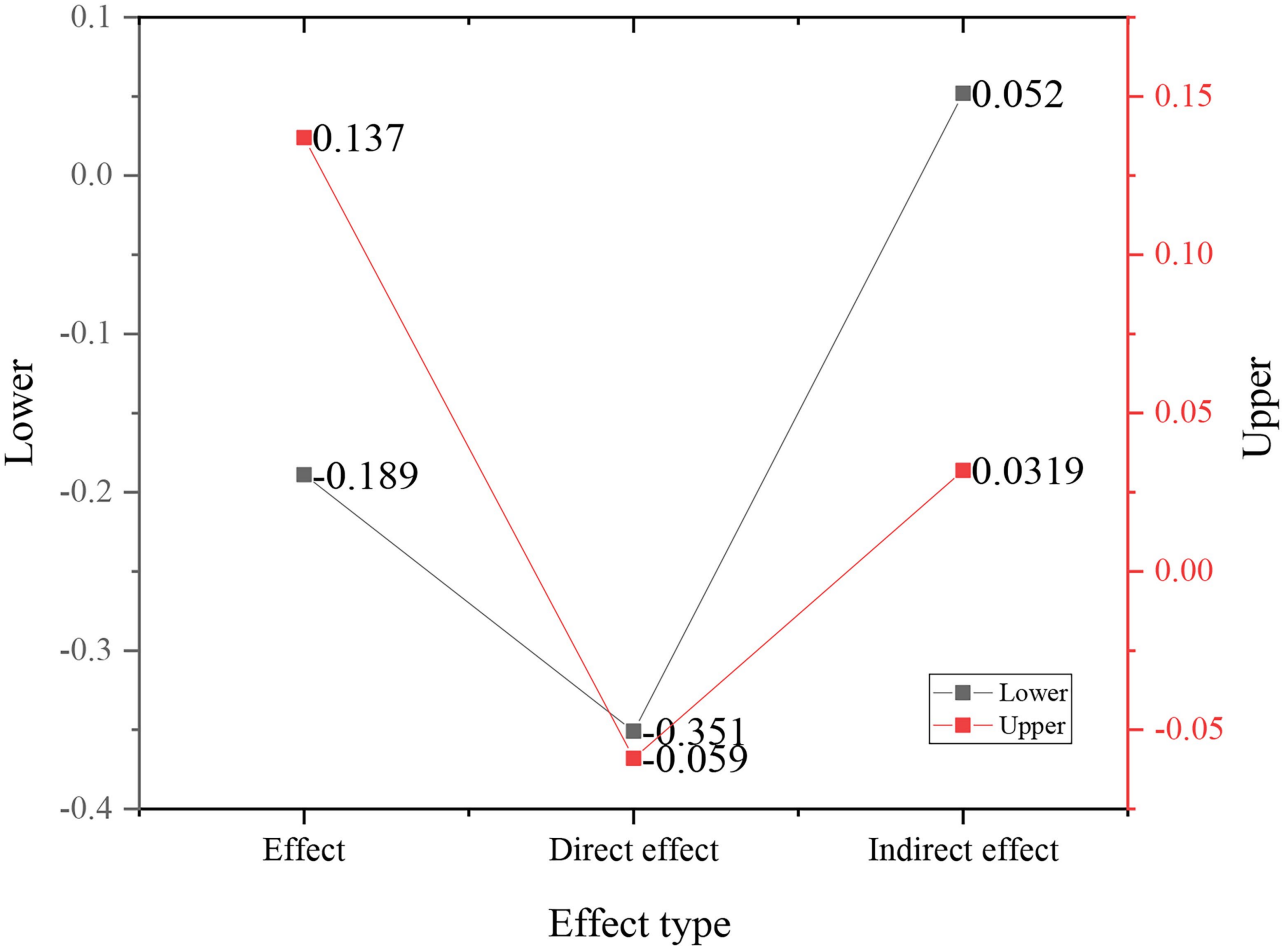
Comparison of deviation calibration.

The comparison of the bottom value and the top value of the deviation calibration results is shown in Figure 7.

According to Figure 7, the upper limit of the direct effect of music professional education on entrepreneurial intention is −0.059, and the lower limit is −0.351. The upper limit of the indirect effect is 0.319, and the lower limit is 0.052, both excluding 0. It is obvious that the direct effect is more significant. It is inferred that the self-efficacy of entrepreneurship should play a certain intermediary effect between music professional education and entrepreneurial intentions. The above T3 is verified.

### Analysis of Gender Regulation

**Figure 8:**
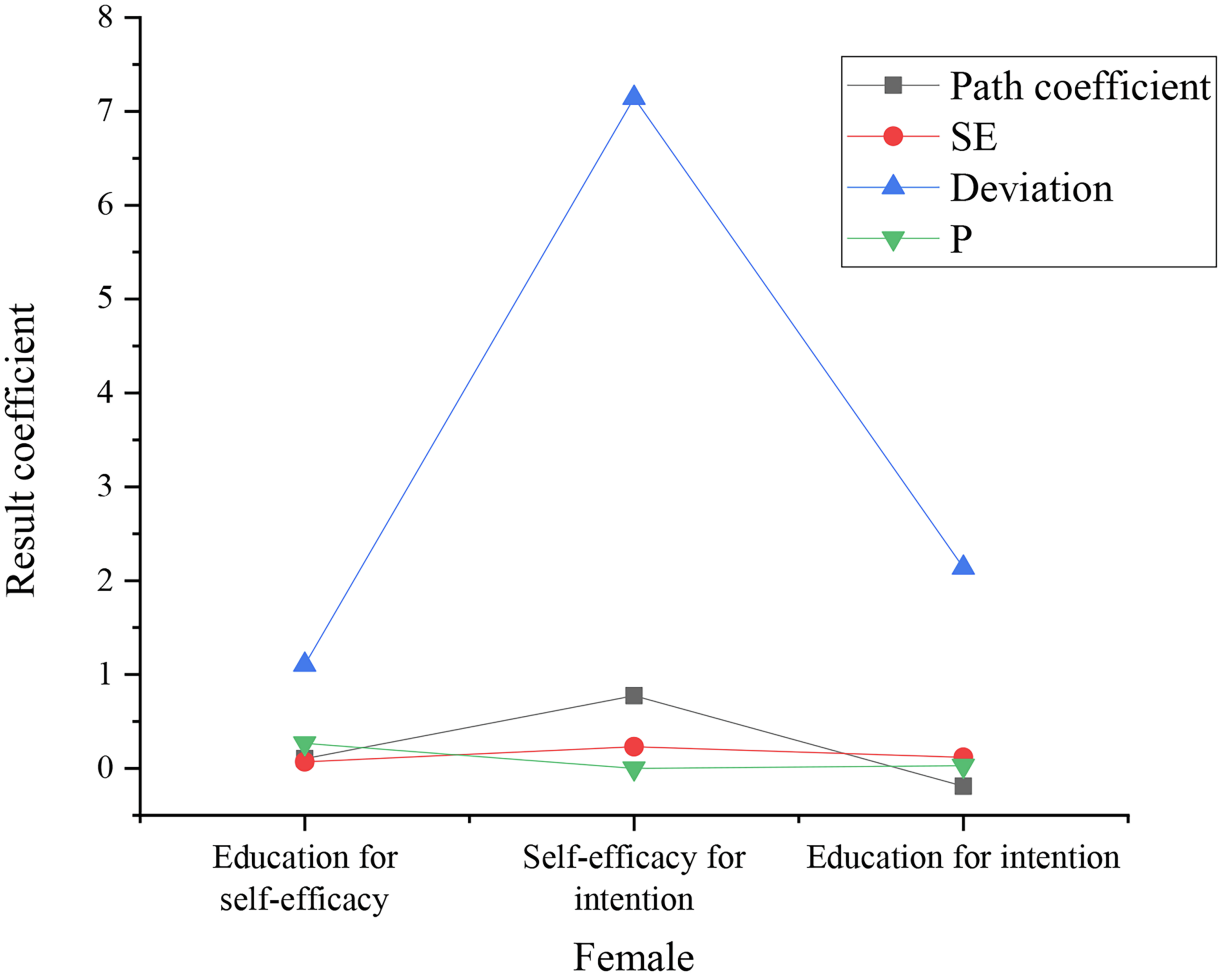
Estimated results of the female sample.

**Figure 9:**
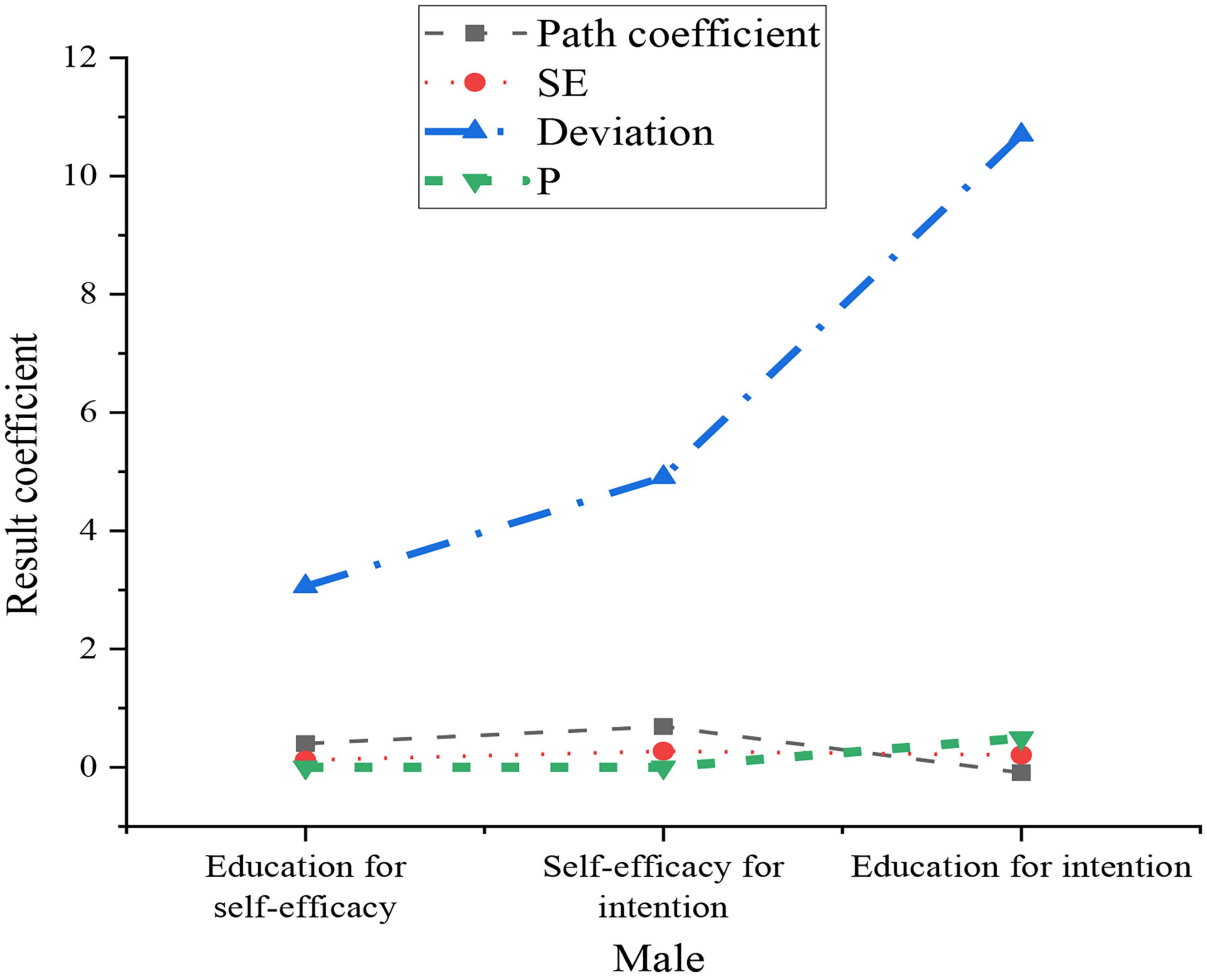
Estimated results of the male sample group.

The subjects of different genders were divided into two groups for the relationship test to analyze the moderating effect of gender. The group estimation results are shown in Figures 8, 9.

In Figure 9, the estimated results of the male group show that the self-efficacy of education is positively correlated with the purpose of self-efficacy, and there is a considerable positive correlation between the purpose of self-efficacy and the purpose of education. Nonetheless, there is an insignificant correlation between educational self-efficacy and educational purpose.

**Figure 10:**
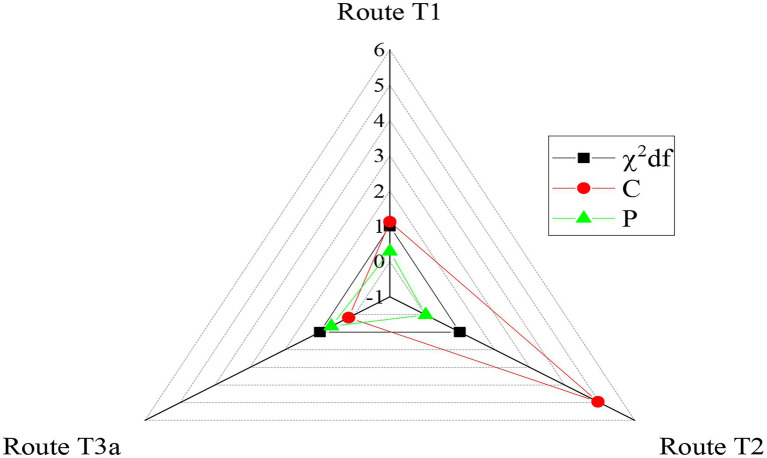
Chi-square test.

The chi-square test of the model is shown in Figure 10.

According to the data obtained from the above research steps, two models, the finite model, and the default model, are further established by using the method of group analysis. The finite model restricts the path coefficients of gender groups. For groups of different genders, the test results are shown in Table 6.

**Table 6:** Equation model fitting exponent.

Adaptation indicators	Coefficient values
χ^2^/df	1.712
GFI	0.868
χ^2^	409.2
NFI	0.768
IFI	0.936
CFI	0.938
RMSEA	0.051

The results show that the constraint model has a fitting degree. There is a significant difference in the chi-square value of the entrepreneurial self-efficacy path in the acceptance of music professional education among different gender groups, while there is a small difference in other paths. The influence of music professional education on the entrepreneurial self-efficacy of men is significantly higher than that of women, so the T4 hypothesis is verified, while T5 is not.

### Discussion and Analysis of Experimental Results

Based on the above experimental data, gender adjustment has a certain influence on the entrepreneurial self-efficacy of music education students and can have a moderating effect on the entrepreneurial intention of students. Entrepreneurship education for students can improve the competitiveness of students and contribute to the overall cultivation of talent in colleges and universities. Compared with female students, male students are more willing to take risks and more likely to obtain achievement motivation. In addition, it is clear that the entrepreneurial environment for women is significantly less favorable than that for men, and they receive far less social support and opportunities. Therefore, the entrepreneurial intention of men tends to be stronger by comparison.

The reform of music teacher education in colleges and universities should closely follow the pace of music education reform in primary and secondary schools. One of the highlights of the 2017 edition of *Music Curriculum Standards for General High Schools* is the addition of core competencies in music disciplines. It is believed that the fundamental value of educating people in contemporary music disciplines should be reflected in three aspects: artistic expression, cultural understanding, and aesthetic perception, reflecting the unique contribution of music discipline education to student growth and lifelong development. In primary and secondary schools, music education aims to cultivate and develop the core literacy of music subjects of students. Therefore, music teachers should deeply understand the connotation and relationship of the core literacy of music disciplines and integrate its main purpose into the whole process and every link of music curriculum learning.

In summary, in the current process of talent innovation training for musicology majors in local colleges and universities, it is necessary to grasp the laws of social development and understand the relevant needs of current talent training. Based on the knowledge system of the music major itself, the advantages of local colleges and universities are combined to create a diversified classroom system. Building on an interdisciplinary and interprofessional basis and expanding students’ cultural horizons can lay a foundation for the improvement of students’ musical literacy and create a variety of teaching methods and curriculum structures.

## Conclusion

Music education in colleges and universities is one of the vital links in training high-quality talent. In recent years, music education in colleges and universities has developed rapidly and has made great achievements. However, there are still many problems with the existing education mode, which is in urgent need of reform. Consequently, questionnaires and stratified sampling were used to construct models and values to verify the relationship and effect of music education, entrepreneurial self-efficacy, and entrepreneurial intention from the perspective of entrepreneurial education as well as the mediating effect. Then, the moderating function of gender in three-dimensional spatial relations was tested. Comprehensive music professional education can contribute to the talent reserve in colleges and universities, and it has a positive impact on college students’ entrepreneurial self-efficacy. Furthermore, entrepreneurial self-efficacy had a positive correlation with entrepreneurial intention, so it played a certain moderating effect between music education and entrepreneurial intention. In the verification, gender regulation played a role in music education’s effect on entrepreneurial self-efficacy. Nevertheless, the mediating effect of gender on the relationship between music education and entrepreneurial intention has not been verified. The effect of gender regulation is insignificant, possibly because women’s present mind is stimulated, and the right to education and concepts are developing toward equality. Some deficiencies in this work are as follows. Firstly, due to limited conditions, only a single region is selected as the research sample, without enough persuasion. Secondly, in the third part of the paper, some conclusions need to be tested in educational practice and to be further improved. Moreover, these existing deficiencies will be improved in subsequent research.

## Data Availability Statement

The raw data supporting the conclusions of this article will be made available by the authors, without undue reservation.

## Ethics Statement

The studies involving human participants were reviewed and approved by Tianjin University of Commerce Ethics Committee. The patients/participants provided their written informed consent to participate in this study. Written informed consent was obtained from the individual(s) for the publication of any potentially identifiable images or data included in this article.

## Author Contributions

The author confirms being the sole contributor of this work and has approved it for publication.

## Conflict of Interest

The author declares that the research was conducted in the absence of any commercial or financial relationships that could be construed as a potential conflict of interest.

## Publisher’s Note

All claims expressed in this article are solely those of the authors and do not necessarily represent those of their affiliated organizations, or those of the publisher, the editors and the reviewers. Any product that may be evaluated in this article, or claim that may be made by its manufacturer, is not guaranteed or endorsed by the publisher.
